# The Portuguese Validation of the Basic Psychological Need Satisfaction and Frustration Scale: Concurrent and Longitudinal Relations to Well-being and Ill-being

**DOI:** 10.5334/pb.252

**Published:** 2016-07-13

**Authors:** Pedro Cordeiro, Paula Paixão, Willy Lens, Marlies Lacante, Koen Luyckx

**Affiliations:** 1 Faculty of Psychology and Educational Sciences University of Coimbra Portugal, PT; 2 KU Leuven, BE

**Keywords:** Self-Determination Theory, Basic Psychological Needs, New Basic Psychological Need Satisfaction and Frustration Scale, Well-being, Emotional Symptomatology

## Abstract

This research comprises two studies based on Self Determination Theory. In Study 1, we translate and examine the factor structure of the Basic Psychological Needs Satisfaction and Frustration Scale (BPNSFS; Chen, Vansteenkiste et al., 2015) in a sample of Portuguese undergraduate students. Further, in Study 2 we used an independent longitudinal sample of 12^th^ grade students to inspect whether the six subscales differently predict adjustment over time. Confirmatory factor analysis showed that a six-factor solution best fitted the BPNSFS data. Subsequent structural equation modelling indicated that the dimensions of need satisfaction and need frustration predicted unique variance in participants’ well-being and ill-being over time, even after controlling for reciprocal and baseline effects. Taken together the findings support the 6-factor multidimensional structure of the BPNSFS and provide extensive support for the distinction between the satisfaction and frustration dimensions of needs, suggesting that they should be measured and interpreted as relatively distinct motivational constructs.

Self-determination theory (SDT; Deci & Ryan, 1985, 2000) is an organismic meta-theory of intrinsic motivation that holds psychological needs as a key psychological construct. Within SDT related to motivation and personality develop from the continued dialectical interplay between the organismic integrative tendencies towards psychological development (Ryan, 1995) and the social-contextual conditions that either support or thwart the satisfaction of needs (Deci & Ryan, 1985).

This innate tendency for growth and optimal integrated functioning is based on the processes of intrinsic motivation and internalization (Deci & Ryan, 2000). Intrinsic motivation describes the natural, basic tendency of individuals to actively engage in interesting and pleasurable activities that promote growth and optimal functioning. Internalization refers to the natural inclination to integrate extrinsic aspects of the social environment in the sense of self (e.g., values, goals, behaviors; Deci & Ryan, 2000).

Intrinsic motivation and internalization develop when significant social contexts provide experiences that satisfy the child’s needs for autonomy (self-endorsement, volition and choice in personal actions), competence (being effective in successful goal attainment) and relatedness (feeling part of mutually gratifying, intimate and accepting relations; Deci & Ryan, 2000; Ryan, 1995).

This integrative growth-oriented process is undermined when individuals develop in social contexts that deprive need satisfaction, or, more importantly, when they are chronically exposed to social environments that actively thwart the satisfaction of the needs (Vansteenkiste & Ryan, 2013). The experience of need deprivation seems to be related to the development of subjective feelings of need dissatisfaction (or absent need satisfaction), whereas the experience of social need thwarting makes individuals become more vulnerable to develop feelings of need frustration (Vansteenkiste & Ryan, 2013), namely feelings of being externally and internally controlled and pressured (autonomy frustration), doubts about one’s efficacy, feelings of failure and inferiority (competence frustration) and experienced relational exclusion and loneliness (relatedness frustration; Bartholomew, Ntoumanis, Ryan, & Thøgersen-Ntoumani, 2011; Deci & Ryan, 1985; Vansteenkiste & Ryan, 2013).

There is an ongoing discussion within SDT whether the subjective experiences of need dissatisfaction and of need frustration correspond both to the negative pole of the need satisfaction continuum, or constitute substantively distinct phenomena, with need frustration being situated on a different motivational continuum (Bartholomew, et al., 2011). Emergent SDT-based research has consistently favored the latter hypothesis. It was found that low scores on psychological need satisfaction predicted lowered well-being (e.g., Ryan, Deci, & Grolnick, 1995), but not ill-being (e.g., Adie, Duda, & Ntoumanis, 2008; Quested & Duda, 2010), whereas the experience of need frustration was uniquely related to ill-being (Bartholomew, et al., 2011; Cordeiro, Paixão, Lens, Lacante, & Sheldon, 2016; Haerens, Aelterman, Vansteenskiste, Soenens, & Petegen, 2015). These associations were consistently reported in a variety of contexts, including physical education (Delrue et al., this issue, Haerens, et al., 2015; Gunnell, Crocker, Wilson, Mack, & Zumbo, 2013), work (e.g., Gillet, Fouquereau, Forest, Brunault, & Colombat, 2011, Van den Broeck, Vansteenkiste, De Witte, Soenens, & Lens, 2010), and interpersonal relations (Costa, Ntoumanis, & Bartholomew, 2014).

Overall there is today consensus that need dissatisfaction captures the feelings of absent need satisfaction (or need dissatisfaction) rather than the active nature and intensity of need frustration (e.g., Costa, Ntoumanis, & Bartholomew, 2014; Vansteenkiste & Ryan, 2013). Thereby, need dissatisfaction and need frustration should be conceptualized as substantively distinct constructs (Bartholomew et al., 2011; Costa, Ntoumanis, & Bartholomew, 2014).

## Empirical Work on Need Satisfaction and Need Frustration

Consistent with the theoretical distinction between need satisfaction and need frustration, new measures of psychological needs have been used that tap into the satisfaction and frustration dimensions of each need (Sheldon & Hilpert, 2012; Chen, Vansteenkiste et al., 2015). Bartholomew and colleagues (2011) developed and validated the Psychological Need Thwarting Scale - PNTS, a 6-factor scale designed to measure the experiences of psychological need satisfaction (3 scales) and need frustration (3 scales) in the sports context. In the original validation studies, the researchers found that the athletes’ perceptions of need satisfaction predicted concurrent vitality and positive affect in sports, whereas need thwarting predicted maladaptive symptoms, such as burnout, depression, and somatic complaints. In a subsequent study using a modified version of the PNTS, Gunnell and collaborators (2013) found that psychological need thwarting (here referred to as psychological need frustration) related uniquely to negative affective states in recreational exercisers above and beyond need satisfaction.

Later on, Sheldon and Hilpert (2012) developed the Balanced Measure of Psychological Needs (BMPN) Scale, a measure of psychological need satisfaction, with three positively-worded scales measuring autonomy, competence, and relatedness satisfaction at the general (instead of domain-specific) level, and three negatively-worded scales measuring need frustration. Similar to Bartholomew and colleagues (2011), Sheldon and Hilpert (2012) provided preliminary empirical evidence for the substantive distinction between the need satisfaction and frustration dimensions at both the factor-analytic and predictive levels, making it clear that the frustration of needs acted as the unique predictor of psychological ill-being.

More recently, Chen and colleagues (2015) developed, in collaboration with Edward Deci and Richard Ryan, the Basic Psychological Need Satisfaction and Frustration Scale (BPNSFS), a measure of the satisfaction and frustration dimensions of the psychological needs. In the original cross-cultural validation studies, carried out in large and culturally diverse student samples, the dimensionality of the BPNSFS was examined as well as the possible unique effects of autonomy, competence, and relatedness satisfaction and frustration on well-being and ill-being. Factor-analytic procedures provided evidence for the hypothesized multidimensional 6-factor structure, which discriminates between the satisfaction and frustration components for each need, with the items being interpreted similarly by participants from diverse cultures. Further, SEM analyses showed that the satisfaction of each of the three needs is associated with well-being (vitality and life satisfaction), whereas the frustration of each of the three needs uniquely relates to ill-being (Chen, Vansteenkiste et al., 2015), not only among individuals who value or desire, but also among those who do not value or desire to get their needs met. Overall, findings support the claim that the psychological needs are universal nutriments for optimal integrated functioning across cultures, and that these needs operate independently of individual differences.

Two reasons justify the continued investigation on the distinct contribution of psychological need satisfaction and frustration to psychosocial outcomes. First, despite the fact that the BPNSFS has been successfully adapted for both western and non-western samples, (e.g., Peru, China, Belgium, US, and South Africa), most studies made use of cross-sectional (e.g., Chen, Van Assche, Vansteenkiste, Soenens, & Beyers, 2015; Chen, Vansteenkiste et al., 2015; Van der Kaap-Deeder et al., 2015) or diary studies (Kindt, Vansteenkiste, Loeys & Goubert, 2016; Mabbe, Soenens, Vansteenkiste, Van der Kaap-Deeder, & Mouratidis, 2016) and only one used an experimental design (Van Petegem, Soenens, Vansteenkiste, & Beyers, 2015). The lack of longitudinal research does not allow to answer the question of whether need satisfaction and frustration have enduring negative relations and bear distinctive effects on adjustment over time. Second, despite the fact that the BPNSFS has been used to examine needs at both the general and the domain-specific levels (including physical education, relationships and teacher training; e.g., Aelterman, Vansteenkiste, Van Keer & Haerens, 2016, Haerens et al., 2015), no studies so far have used the BPNSFS to assess adolescent adjustment during potential stressful periods for career identity construction.

### The present research

The aims for the current research are to develop and validate the Portuguese version of the BPNSFS and examine its concurrent and longitudinal relations to both well-being and ill-being. In Study 1 we translate the BPNSFS into Portuguese and examine the (construct validity) of the scale in a sample of Portuguese undergraduate students. With this procedure we expect to bring additional cross-cultural validation data for the invariance of the multidimensional six-factor model of scales validated in the original studies (Chen, Vansteenkiste et al., 2015). In Study 2 we examined, using a longitudinal design, whether 12^th^ grade students’ baseline feelings of need satisfaction and frustration predict unique changes in well/ill-being when they are expected to decide whether they want to continue their studies or join the labour market.

## Study 1

The purpose of Study 1 was to provide initial evidence for the validity of the translated BPNSFS. In a first step, we translated the scale into Portuguese, following the back-translation procedures proposed by Hambleton (2001). As in the original studies (Chen, Vansteenkiste et al., 2015) the internal structure of the BPNSFS was inspected in a two-step approach. In Step 1, we fitted three competitive models to the BPNSFS data. The first Model replicates the original 6-factor solution of scales proposed by Chen and colleagues (2015), with the BPNSFS items being organized in a structure of 6 latent variables, corresponding to the scale scores of the satisfaction and frustration dimensions of each of the three need subscales. This model was compared with two competitive and theoretically reasonable factor solutions: a) a two-factor higher order model with two latent 2^nd^ order factors of need satisfaction and frustration, each indicated by three first-order factors of autonomy, competence and relatedness needs, and b) a “common-trait”, “common-method” model, in which each of the items loads simultaneously on its respective latent factor (measuring autonomy, competence and relatedness needs) and on the “satisfaction” and “frustration” method factors.

The fit of the measurement model was checked using conventional fit indices: the Chi square (*X^2^*) statistics, the Standardized Root Mean Square Residual (*SRMR*), the Comparative Fit Index (*CFI*) and the Root Mean Squared Error of Approximation (*RMSEA*). Model fit followed the cut-off values of .09 for *SRMR*, .06 for *RMSEA*, and .90, or above, for *CFI* (Hu & Bentler, 1999). Items exhibiting poor factor loadings (λ*i* < 0.4) or large cross-loadings (Modification Indices) were excluded from further analyses.

In a second step we performed a multigroup Confirmatory Factor Analysis (CFA; Byrne, 2010) on the BPNSFS to examine the invariance of the six-factor model across age. This procedure was considered necessary given that the age range in the sample is quite broad (18 to 37 years old). A sequential model testing approach was followed, with two models specified in AMOS 20.0. An unconstrained model (where factor loadings were allowed to vary) was compared to two increasingly constrained models, where factor loadings (measurement equality model), factor variances and co-variances (structural parameters model) and intercepts were set equal across age. Model invariance was indicated by the combined χ^2^ difference test (Byrne, 2010).

## Method

### Participants

As in the original studies (Chen, Vansteenkiste et al., 2015; study 2), in Study 1 we examined the psychometric properties of the BPNSFS in a sample of undergraduate students (*N* = 417; male 41%; female 59%), aged between 18 and 37 years (mean age = 20.41 years; SD 0.47). Participants attended the following majors: Psychology (1^st^ year *N* = 144[34.5%]; 2^nd^ *N* = 68 [16.3%]; 3^rd^ 38[9.1%]), Social Work (1^st^ year *N* = 32[7.7%], Journalism (3^rd^ year *N* = 53 [12.7%]) and Mechanical Engineering (2^nd^ year, *N* = 82 [19.7%]) at the universities of Lisbon (*N* = 141 [33.8%]), Porto (*N* = 38 [9.1%]), Coimbra (*N* = 103 [24.7%]), and at the Polytechnic Institute of Leiria (*N* = 135 [32.4%]).

### Measures

***Psychological Need Satisfaction and Frustration.*** Participants completed the Portuguese version of the Basic Psychological Need Satisfaction and Frustration Scale (BPNSFS; Chen, Vansteenkiste et al., 2015). The original version of the scale, worded in Dutch but also available and validated in English by the authors, was translated into Portuguese by two Portuguese-speaking researchers fluent in English, in close collaboration with a native English researcher. Next, the items were translated back into English by a professional translator. Both original and back translated versions were checked for accuracy and whenever discrepancies were found, they were discussed until consensus was achieved (Hambleton, 2001). In a final procedure, the BPNSFS was pilot-tested in a small sample of undergraduate students (N = 17; gender: 11 girls; 6 boys; age range 18-23 years old). The items were unequivocally understood by the participants. Thereby no further changes were made to the items.

The original 24-item BPNSFS is organized in a multidimensional structure of six scales. Three of these scales tapped into experiences of satisfaction of the three psychological needs for autonomy, (e.g., “I feel a sense of choice and freedom in the things I undertake”), competence (e.g., “I feel confident that I can do things well”), and relatedness (e.g., “I feel close and connected with other people who are important to me”). The three remaining scales assess the level of frustration of each of the needs, that is, autonomy (e.g., “I feel pressured to do too many things”), competence (e.g., “I feel insecure about my abilities”), and relatedness (e.g., “I feel the relationships I have are just superficial”). Items are rated on a 5-point Likert scale, ranging from 1 (*“Completely untrue”*) to 5 (“*Completely true*”). In the original studies the six scales show an adequate internal consistency (Chen, Vansteenkiste et al., 2015), with Cronbach’s alphas ranging across samples between .73 and .89 for the satisfaction subscales and between .64 and .86 for the frustration subscales (Chen, Vasteenkiste et al., 2015). In the current sample the Cronbach alphas ranged between .70 for autonomy frustration and .87 for competence satisfaction (see Table 1)

**Table 1:** BPNSFS. Global Fit Indices for the Measurement Models Tested. Multiple-group Analysis.

Confirmatory Factor Analysis	χ^2^	*df*	*CFI*	*RMSEA*	*SRMR*	*AIC*	*Comparison of models*		

							**Δχ^2^**	**Δ*df***	***p-value***

**Measurement Models**									
6-factor model (Sample1)	519.13	237	.95	.05	.06	645.13			
3+2 factor model (Sample 1)	546.30	227	.94	.06	.08	694.30			
2-factor model (Sample 1)	852.81	235	.89	.08	.07	982.81			
6-factor model (Sample2)	245.40	120	.97	.05	.04	347.40			
2-factor model (Sample 2)	298.93	128	.95	.05	.05	384.93			
**Multiple‑group analysis for age**									
Unconstrained	397.43	240	.96	.04	.06				
Measurement weights	410.60	252	.96	.40	.70		Δ*χ^2^* = 13.17	Δ*df* = 12	**.36**
Structural covariances	444.92	273	.96	.40	.10		Δ*χ^2^* = 47.49	Δ*df* = 33	**.05**
Measurement residuals	553.18	291	.93	.05	.10		Δ*χ^2^* = 155.76	Δ*df* = 51	.01

*Note1*: χ^2^ = qui-square; *CFI* = comparative fit index; *RMSEA* = Root Mean Square Error of Approximation; *SRMR* = Standardized Root Mean Square Residual; *AIC* = Akaike Information Criterion; *p* < 0.0001; Δχ^2^ = Qui-Square Difference; Δ*CFI* = Difference in Comparative Fit Index; “**Bold**” values indicate non-significant changes in model fit.

### Procedure

The final version of the BPNSFS was administered to the participants after approval from the ethical committee of the Faculty of Psychology and Educational Sciences of the University of Coimbra. Prior to scale administration, active informed consent from students was obtained. Students completed the questionnaires in approximately 25 minutes. Participation was voluntary. Anonymity was also guaranteed, and no credits were received for the involvement in the study.

## Results

***CFA*.** Table 1 summarizes the goodness-of-fit estimates for Study 1. Initial estimation of the 6-factor model yielded a good fit to the data: χ^2^ (237) = 519.128 *p*<.001, CFI = .945, RMSEA = .053 *p* [RMSEA ≤ 0.05], SRMR = .054. The scales expressed good internal consistency that ranges between .70 for autonomy frustration and .85 for competence and responsiveness satisfaction. The standardized factor loadings of the items ranged between .40 and .89 (*p* < .001) with a mean of .71.

***Model invariance*.** The equivalence of the six-factor model of scales was demonstrated across younger and older student age groups: The unconstrained model yielded an adequate fit to the data, with all factor loadings being statistically significant. The unconstrained and the constrained models were not significantly different, providing evidence for the invariance of the model across age (χ*^2^* < .001) in terms of factor loadings, structural covariances. Hence, despite the high age range of the participants, the sample is generally homogeneous with respect to the constructs measured.

***Alternative models*.** The six-factor solution was, then, compared with two alternative measurement models: a two-factor higher order model measuring the satisfaction and frustration of psychological needs (as in Haerens et al., 2015), and a common-trait, common-method model that is intended to detect possible method effects (see also Sheldon & Gunz, 2009; Sheldon & Hilpert, 2012). The two-factor higher order model yielded a marginal fit to the data χ^2^ (235) = 852.810 *p* <.001, CFI = .887, RMSEA = .079 *p* [RMSEA ≤ 0.05], SRMR = .070. Furthermore, the 3x2 common-trait, common-method model (see Sheldon & Gunz, 2009; Sheldon & Hilpert, 2012) yielded a good fit to the data χ^2^ (227) = 546.295 *p*<.001, CFI = .937, RMSEA = .058, *p* [RMSEA ≤ 0.05], SRMR = .077 with items loading simultaneously on their respective latent need factor and on the “satisfaction” or “frustration” method factor. However, the lowest AIC obtained for the 6-factor solution (AIC = 645.128) supports this factorial solution as the preferred one to interpret the theoretical structure of the BPNSFS data.

## Study 2

**Figure 1:**
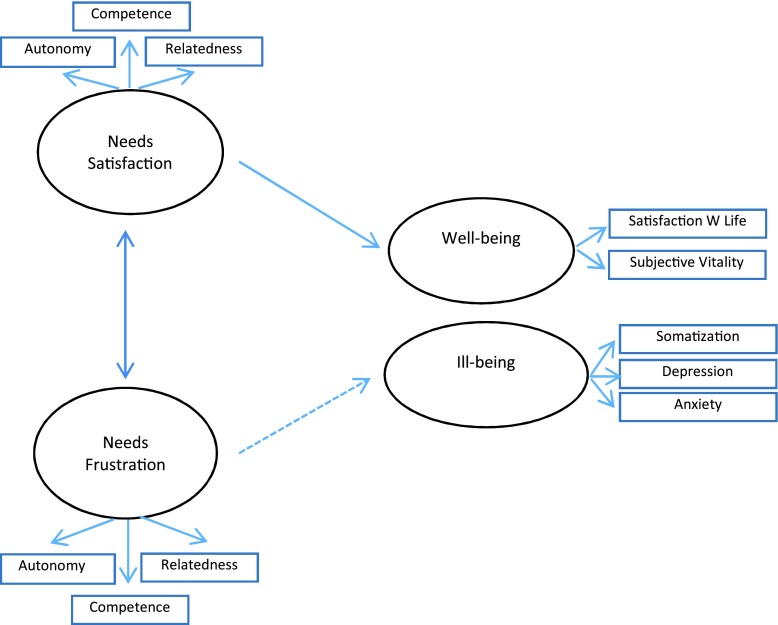
Model Overview. SEM Model 1 and 2. *Note:* Solid line: Model 1; Dotted line: Model 2.

**Figure 2:**
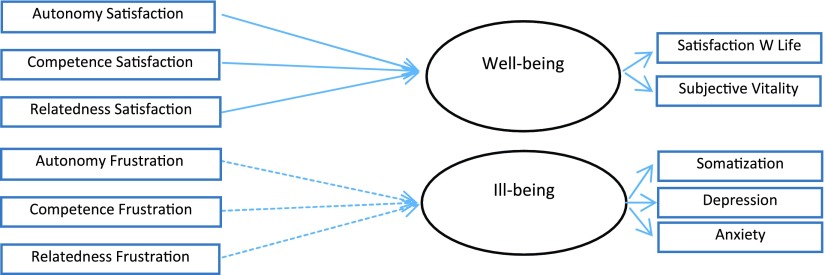
Model Overview. SEM Model 3 to 5. *Note:* Solid line: Model 3; Dotted line: Model 4. Model 5 specifies the six predictors simultaneously.

In Study 2 (Figures 1 and 2) we examined, using a longitudinal study design, whether the satisfaction and frustration of psychological needs predict unique changes in the students’ well and ill-being. Data were collected in two points of time: Time 1 (October, 2013), and Time 2 (July, 2014), after students ended the mandatory exams to access university. We used a stepwise approach. In Step 1 we looked at overall need satisfaction and need frustration and in Step 2 we decomposed these overall measures to examine the unique contribution of the three needs to the outcomes. In Step 1 we specified two separate structural models with pathways from T1 need satisfaction to T2 well-being (Model 1) and from T1 need frustration to T2 ill-being (Model 2), controlling for baseline effects of T1 well-being and T1 ill-being. The trajectories identified were flagged significant at *p* < 0.05. Gender was modeled as a single indicator with error variance fixed to 0. We expected that averaged need satisfaction score at T1 would positively predict changes in well-being at T2, controlling for baseline well-being, whereas the averaged need frustration score at T1 would positively predict changes in ill-being at T2, controlling for baseline ill-being at T1 (*Hypothesis 1*). To examine this prediction we ran Structural Equation Modeling analyses where T1 need satisfaction and need frustration entered simultaneously as predictors of T2 well-being and ill-being, controlling for autoregressive effects of well-being and ill-being at T1.

In Step 2 we broke down the composite scores of need satisfaction and need frustration into six distinct indicators to examine whether autonomy, competence and relatedness need satisfaction and frustration predicted unique variance in well-being and ill-being outcomes. We performed SEM analysis, following a stepwise approach (see Chen, Vansteenkiste et al., 2015). Firstly the three T1 need satisfaction measures were entered as simultaneous predictors of T2 well-being, after controlling for baseline T1 well-being. Secondly the three T1 need frustration scores were entered as simultaneous predictors of T2 ill-being, controlling for baseline T1 ill-being. We expected that the satisfaction of the three needs predicts unique changes on T2 well-being controlling for baseline T1 well-being, but also that the frustration of each need predicts unique changes on T2 ill-being, controlling for baseline T1 ill-being (*Hypothesis 2*).

## Method

### Participants

At Time1, participants of Study 2 consisted of 755 Portuguese 12^th^ grade students (Girls *N* = 455 [60.3%]; Boys *N* = 300 [39.7%]), aged between 16 and 22 years, with a mean age of 17.36 years (*SD* = 0.89)^1^. The students targeted attended scientific-humanistic (*N* = 652 [86.4%]) or technical-vocational courses (*N* = 103 [13.6%]). At T2, a sample of 417 Portuguese students (278 girls [60.2%] and 184 boys [39.8%], aged between 16 and 22 years, with a mean age of 17.12 years (*SD* = 0.92), completed the questionnaires. Participants were free to participate in the study at both T1 and T2. Around 38% of the participants dropped out the study at T2 because they were having scheduled exams, were randomly absent from the class, or were involved in apprenticeship curricular activities or in sports competitions. These students did not differ in gender, age or on any psychological measure assessed at T1. The questionnaires were administered after we obtained the necessary permissions from the Portuguese Bureau of Basic and Secondary Education and from the ethical committee of the Faculty of Psychology and Educational Sciences of the University of Coimbra. Prior to scale administration, active informed consent was obtained from students who participated in the study and passive informed consent was obtained from the parents of underage students (< 16 years).

### Measures

***Psychological Need satisfaction and Frustration*.** Participants completed the Portuguese version of the BPNSFS (Chen, Vansteenkiste et al., 2015) as above described.

***Well-Being.*** The hedonic component of subjective well-being was measured with the Satisfaction With Life Scale – SWLS developed by Diener, Emmons, Larsen, and Griffin (1985; e.g., “I am satisfied with my life”; Portuguese version, Simões, 1992). The Cronbach’s alpha was of .87 in the original study and .77 in the Portuguese translation studies. In addition, the eudaimonic component of well-being (Ryan & Deci, 2001) was assessed via the Subjective Vitality Scale (Ryan & Frederick, 1997, α = .84; Portuguese version, Lemos, Gonçalves, & Coelho, 2011; α = .86). The SV scale is a 7-item measure developed to evaluate how alive and alert people have been feeling during the last month (e.g., “I feel alive and vital”). The items were rated on a 5-point Likert scale, ranging from 1 (*“Not at all true”*) to 5 (“*Very true*”). The Cronbach alpha reported for the Subjective Vitality Scale was .84. The internal consistency of the scales was .80 for SWL and .86 for the Subjective Vitality Scale. Items loaded above .61.

***Ill-Being*.** The Portuguese version of the 18-item Brief Symptom Inventory (Derogatis, 2001; Canavarro, 2007) was used to assess ill-being. The BSI-18 assesses the psychopathological symptoms of anxiety (e.g., “Feeling tense or keyed up”), depression (e.g., “Feeling lonely”), and somatization (e.g., “Pains in heart or chest”). A General Severity Index can also be obtained. The items are rated on a 4-point Likert scale of distress, ranging from 0 (*“Not at all”*) to 4 (“*Extremely*”). The internal consistency reported for the 3 subscales ranged between .62 and .80 in the Portuguese version (Canavarro, 2007). In the current study we used the General Severity Index as an indicator of ill-being, obtained from averaged total scores on the 18 items. Good internal consistency was observed for the General Severity Index items (α = .87)

## Results

***Sample attrition*.** We detected 33% of missing data from T1 to T2. We looked for patterns in missing data we ran the Expectation-Minimization (EM) algorithm in SPSS. A non-significant Little’s MCAR test, χ*^2^*(678) = 600.498, *p* = .985, indicated that the data were missing at random (Little, 1988). Then we examined the differences between the data collected at Time 1 and Time 2. We computed the mean scores of well-being and ill-being for both groups of respondents and carried out between group *t*-tests. The groups were not significantly different (*t* < 1.5, *p* > 0.10), thus suggesting that no significant selection biases were found in the dataset. Missing data were, then imputed using the full information maximum likelihood technique that is available in AMOS 20.0 (see Enders & Bandalos, 2011).

***Descriptive Statistics and Correlations*.** Table 2 provides descriptive statistics and correlations for the study variables. In Sample 2, need satisfaction scores positively associate between themselves, positively relate to satisfaction with life and subjective vitality and negatively relate to anxiety, depression and somatization. On the other hand, need frustration indicators were positively associated to each other, and they were also positively related to anxiety, depression and somatization, but negatively related to satisfaction with life and subjective vitality scores. Moderate-to-high correlations were observed between subjective vitality and satisfaction with life, as well as between anxiety, depression and somatization.

**Table 2:** *BPNSFS. Means, Standard Deviations, Range and Internal Consistency of the Variables* (Study 1).

Variables	N	Minimum	Maximum	Mean	SD	Alpha	Zero-order correlations												

Autonomy Satisfaction	417 (755)	1.67 (1.00)	6.00 (6.00)	4.87 (4.58)	0.82 (.90)	.76 (76)	1	2	3	4	5	6	7	8	9	10	11	12	13
Autonomy Frustration	417 (755)	1.00 (1.00)	5.00 (6.00)	2.20 (2.42)	0.92 (.97)	.85 (70)	1	–.39^**^	.51^**^	–.38^**^	.51^**^	–.32^**^	.46^**^	.36^**^	–.15^**^	–.32^**^	–.12^**^	.80^**^	–.43^**^
Competence Satisfaction	417 (755)	1.00 (1.00)	6.00 (6.00)	4.83 (4.72)	0.88 (.83)	.85 (73)	–.50^**^	1	–.28^**^	.50^**^	–.47^**^	.55^**^	–.32^**^	–.28^**^	.28^**^	.43^**^	.21^**^	–.47^**^	.80^**^
Competence Frustration	417 (755)	1.00 (1.00)	6.00 (6.00)	2.13 (2.50)	1.04 (1.0)	.74 (73)	.62^**^	–.39^**^	1	–.58^**^	.42^**^	–.29^**^	.41^**^	.37^**^	–.23^**^	–.37^**^	–.19^**^	.81^**^	–.46^**^
Relatedness Satisfaction	417 (755)	1.25 (1.75)	6.00 (6.00)	5.25 (5.16)	0.78 (.80)	.78 (73)	–.46^**^	.46^**^	–.72^**^	1	–.35^**^	.56^**^	–.40^**^	––.34^**^	.43^**^	.58^**^	.34^**^	–.54^**^	.84^**^
Relatedness Frustration	417 (755)	1.00 (1.00)	6.00 (5.75)	1.80 (1.97)	0.88 (.98)	.80 (70)	.63^**^	–.47^**^	.59^**^	––.49^**^	1	–.60^**^	.39^**^	.33^**^	–.17^**^	–.37^**^	–.15^**^	.79^**^	–.55^**^
Satisfaction With Life	417 (755)	1.00 (1.00)	6.00 (5.00)	1.96 (2.10)	0.79 (.93)	.85 (.82)	–.50^**^	.51^**^	–.56^**^	.61^**^	–.75^**^	1	–.33^**^	–.27^**^	.27^**^	.45^**^	.28^**^	–.51^**^	.84^**^
Vitality	417 (755)	1.00 (1.00)	7.00 (5.00)	1.97 (2.12)	0.87 (.93)	.89 (.92)	.49^**^	–.47^**^	.48^**^	–.50^**^	.47^**^	–.42^**^	1	.49^**^	–.31^**^	–.53^**^	–.20^**^	.44^**^	–.36^**^
Anxiety	417 (755)	1.00 (1.00)	5.00 (5.00)	1.69 (1.63)	0.74 (.70)	.70 (.70)	.44^**^	–.28^**^	.54^**^	–.54^**^	.49^**^	–.41^**^	.53^**^	1	–.28^**^	–.43^**^	–.26^**^	.53^**^	–.42^**^
Depression	417 (755)	1.00 (1.00)	5.00 (5.00)	4.99 (4.83)	0.70 (.65)	.87 (.83)	–.31^**^	.34^**^	–.39^**^	.49^**^	–.33^**^	.40^**^	–.36^**^	–.39^**^	1	.66^**^	.69^**^	–.23^**^	.39^**^
Somatization	417 (755)	1.00 (1.00)	7.00 (5.00)	2.02 (2.44)	0.79 (.81)	.82 (.80)	–.40^**^	.38^**^	–.52^**^	.65^**^	–.47^**^	.51^**^	–.56^**^	–.56^**^	.73^**^	1	.49^**^	–.44^**^	.58^**^
General Satisfaction	417 (755)	1.78 (2.25)	6.00 (6.00)	3.74 (3.46)	0.86 (.95)	.90 (81)	–.14^**^	.21^**^	–.25^**^	.32^**^	–.22^**^	.27^**^	–.26^**^	–.23^**^	.63^**^	.49^**^	1	–.20^**^	.33^**^
General Frustration	417 (755)	1.00 (1.00)	4.89 (5.50)	4.67 (3.90)	1.15 (.83)	.88 (83)	.85^**^	–.52^**^	.87^**^	–.66^**^	.85^**^	–.69^**^	.55^**^	.58^**^	–.40^**^	–.54^**^	–.24^**^	1	–.59^**^

*Note*: values under brackets refer to the descriptives at T1 high school students **.*p* < .01 level ; *. *P* < 0.05. *Note:* SD = Standard Deviation; Intercorrelations, Mean and SD at the lower and upper diagonal refer respectively at the samples of undergraduate (N = 417) and high school students at T1 (N = 755), respectively.

***Gender Effects*.** A two-way MANOVA was conducted to examine whether gender was related to any of the study variables. The results indicated the significant multivariate effect of gender (Wilk’s Λ = .93, *F* [1, 463] = 11.47, *p* < .01, multivariate η^2^ = .07). Specifically, girls scored higher on anxiety (*M* = 2.29, *SD* = 0.53), depression (*M* = 2.20, *SD* = 0.54), and somatization (*M* = 1.74, *SD* = 0.40) than boys (*M* = 1.81, *SD* = 0.70; *M* = 1.94, *SD* = 0.66; and 1.45, *SD* = 0.49, respectively). We checked for interaction effects, but gender was not found to be a significant moderator of anxiety, depression and somatization. Based on these gender differences, we controlled for gender in the primary SEM analysis.

***Predictive Validity.** SEM* analyses were used to examine the structural relations specified. We first examined need satisfaction and need frustration at T1 as predictors of both well-being and ill-being at T2 not controlling for stability effects (Model 1: χ^2^ (34) = 121.95 *p* <.001, CFI = .958, RMSEA = .082 *p* [RMSEA ≤ 0.05], SRMR = .024). Findings show that need satisfaction positively predicts T2 well-being (β = .45, *p* < .001), but it could not predict changes in T2 ill-being (β = .08, *p* >*.05*). In a different pattern, need frustration predicted both positive changes in ill-being (β = .50, *p* < .001) and negative changes on well-being (β = .24, *p* < .001) at T2. Next, we controlled for baseline stability in well-being (Model 2b χ^2^ (7) = 4.95 *p* <.001, CFI = .999, RMSEA = .001 *p* [RMSEA ≤ 0.05], SRMR = .009) and ill-being (Model 2b χ^2^ (7) = 4.95 *p* <.001, CFI = .999, RMSEA = .001 *p* [RMSEA ≤ 0.05], SRMR = .009). Findings show that T1 need satisfaction remained as a positive predictor of T2 well-being (β = .13, *p* < *.05*), while T1 need frustration remained as a positive predictor of T2 ill-being (β = .20, *p* < .001). High within-time stability coefficients were obtained for T2 wellbeing (β = .75, *p* < .001) and T2 ill-being (β = .71, *p* < .001). We further examined whether the satisfaction of the three separate needs of autonomy, competence and relatedness predicted well-being and whether the three need frustration variables predicted ill-being at T2 not controlling for stability effects (Model 3; χ^2^ (26) = 75.515 *p* <.001, CFI = .976, RMSEA = .071 *p* [RMSEA ≤ 0.05], SRMR = .029). Findings show that the satisfaction of autonomy (β = .25, *p* < .001), competence (β = .19, *p* < .001), and relatedness (β = .19, *p* < .001) predicted T2 well-being, whereas only the frustration of competence (β = .41, *p* < .001) and relatedness (β = .26, *p* < .001) predicted ill-being at T2. Finally, we controlled for baseline well-being (Model 4a; χ^2^ (6) = 18.004 *p* <.001, CFI = .986, RMSEA = .072 *p* [RMSEA ≤ 0.05], SRMR = .024 and for baseline ill-being (Model 4b χ^2^ (14) = 38.857 *p* <.001, CFI = .986, RMSEA = .068 *p* [RMSEA ≤ 0.05], SRMR = .042) in two separate analyses. It was found that only T1 autonomy satisfaction predicted T2 well-being, (β = .16, *p* < .05), whereas only T1 competence frustration predicted unique variance of T2 ill-being (β = .12, *p* < .05).

## Discussion

Previous studies of the BPNSFS used cross-sectional designs to examine the effects of psychological needs on life satisfaction, vitality and depressive feelings (e.g., Brenning et al., 2015; Chen, Van Assche, et al., (2015) Chen, Vansteenkiste et al., 2015). Despite the promising findings of these previous studies, cross-sectional research makes it difficult to probe into the unique predictive role of psychological need satisfaction and frustration on psychological outcomes across time. Moreover, although these studies covered reasonably large samples of adults and adolescents from diverse cultural backgrounds, including South African adolescents, University students from USA, China, Belgium and Peru, Chinese migrant workers or European pregnant women, the Portuguese context had not yet been targeted. Aligned with these cross-cultural validation studies we carried out the first study examining the concurrent and longitudinal relations of the BPNSFS to well-being and ill-being experienced when Portuguese high school students have to decide whether they intend to continue their studies in higher education or join the labour force. We conducted two separate studies. In Study 1 we translated the BPNSFS into Portuguese and examined the construct validity of the scales in a sample of Portuguese undergraduate students. In Study 2 we used a longitudinal research design to examine the unique contribution of need satisfaction and frustration to predict changes in 12th grade students’ well-being and ill-being over time.

Study 1 findings proved that the six-factor model that distinguishes the satisfaction and frustration dimensions of need was the most valid to interpret the dimensionality of the 24-item Portuguese version of the BPNSFS. This result, along with the good internal consistency obtained for the six scales, replicate the results obtained in the original studies (Chen, Vansteenkiste et al., 2015) and suggest, in line with recent SDT theory (Vansteenkiste & Ryan, 2013) and research (e.g., Bartholomew et al., 2011; Cordeiro, et al., 2016; Quested & Duda, 2010) that need satisfaction and frustration scales represent distinct constructs and should be measured separately.

Study 2 findings provide partial support for our hypotheses. In support of Hypothesis 1, the results show that, after controlling for baseline well-being, T1 need satisfaction predicts changes in well-being, but not in T2 ill-being. Symmetrically, after controlling for baseline ill-being, T1 need frustration predicts unique variance in T2 ill-being, but not on T2 well-being. This particular finding adds to previous SDT theory and research (Bartholomew et al., 2011; Quested & Duda, 2010; Vansteenkiste & Ryan, 2013) showing that need satisfaction and frustration have distinct prospective effects on well-being and ill-being (e.g., Bartholomew et al., 2011; Cordeiro, et al., 2016; Vansteenkiste, Lens, Soenens, & Luyckx., 2006).

There is an absent (negative) relation between need satisfaction and ill-being. Indeed, ill-being was uniquely predicted from experiences of need frustration, but not from experiences of low needs satisfaction. In subsequent analyses, the unique contribution of each of the three needs was tested to explain well-being and ill-being at T2. In partial support for Hypothesis 2, findings show that, after controlling for baseline well-being, T1 autonomy satisfaction was a unique predictor of T2 well-being.

A different pattern was found for need frustration. After controlling for baseline ill-being, only T1 competence frustration predicted T2 ill-being. We can only speculate about these findings. On a conceptual level they favor the SDT assertion that experiences of psychological need satisfaction and frustration have distinct unique effects on motivational criteria (Deci & Ryan, 2000; Vansteenkiste & Ryan, 2013). These findings are in line with previous research (e.g., Chen, Vansteenkiste et al., 2015; Costa, Ntounamis & Bartholomew, 2014) showing the importance of separately measuring and examining the predictive value of needs satisfaction and frustration. Furthermore, the results obtained in Study 2 point out the distinct unique contribution of autonomy satisfaction for well-being and of competence frustration for ill-being. Such pattern of results provided further support for the substantive distinction between the satisfaction and frustration components of needs. They also suggest that feeling vital and satisfied with life during career preparation periods is closely related to the extent to which students experience a perceived internal locus of causality, volition and the possibility of choice in personal actions. On the other hand, the frustration of competence seems to play a unique effect on maladjustment. Indeed, a career pathway is a highly selective, competence-focused, achievement-oriented process that is mainly dependent on academic success. In fact, in the Portuguese educational system students’ decisions are largely dependent upon a mean average score that is calculated both from the mean grade average in secondary education and the results of the national exams that students must perform to access higher education. In this achievement-based context, the perceived (in-)ability to perform well at high school might determine whether students experience well-being or ill-being during this period of critical career decision-making. For instance, for students who feel their need for competence frustrated, studying for the national exams would possibly activate failure-oriented thoughts related to the lack of personal competence and ability to perform well in the task, which, subsequently, would increase their levels of anxiety or depressive feelings.

In sum, contexts might differentially trigger (or activate) need-relevant appraisals of competence that, in turn, determine adaptive or maladaptive adjustment for significant career challenges. From this point of view, competence satisfaction could be considered a protective factor of well-being and need frustration an important risk factor for various maladaptive outcomes, including ill-being (Bartholomew et al., 2011), bulimic symptoms (Verstuyf et al., 2013) and oppositional defiance (Chen et al., this issue). Additional research is, however, needed to support this argument. For instance, future research would need to focus on both contextual antecedents (e.g., autonomy support; Van de Berghe et al., 2013) and personality antecedents (e.g., fear of failure; Michou et al., this issue) of dynamics of both need satisfaction and need frustration (Vansteenkiste, 2016, personal communication).

### Limitations

This research has explored the longitudinal associations between the satisfaction and frustration of psychological needs and adjustment across the transition from high school to higher education. Furthermore, it has uncovered the individual contributions and salience of the satisfaction/frustration of autonomy, competence, and relatedness needs to (mal) adjustment. Although this study extended previous research on this topic, a number of limitations are noteworthy. First, we based our conclusions on samples of undergraduate and high school students, an aspect that limits the generalization of the findings. This is relevant because we suspect that students scoring high on need frustration have already dropped-out of the school system. Future studies should target students at an earlier age and/or enrolled in different educational systems, including apprenticeship systems. We also based our conclusions on a single informant, what might have artificially inflated the relations between the constructs due to shared method variance (Podsakoff, MacKenzie, Lee, & Podsakoff, 2003). Future research should include the perceptions of educators (teachers and parents) of students’ adjustment over time. Finally, despite being longitudinal, our research was correlational in nature. To conduct a more complete study of the role of need frustration in the determination of maladjustment, experimental studies should be carried out (Costa, Ntounamis & Bartholomew, 2014). Thus, the replicability of the findings and the generalizability to other target groups has yet to be demonstrated. To ascertain the robustness of these links, future research should also attempt to examine the specific role played by need frustration on the development of “dark” trajectories (Deci & Ryan, 2000) of human functioning in different life domains (McDonough & Crocker, 2007).

## Conclusion

This research extends previous SDT-based studies in two ways. First, it has provided a cross-cultural replication of the 6-factor solution proposed for the BPNSFS, providing additional evidence for the substantive distinction of the need satisfaction and need frustration components of needs. Second, it provided further insights into the motivational roots of (mal)adjustment across a major educational transition. The findings encourage the change from a tradition of research deducing need frustration from low scores on need satisfaction, towards an approach that examines the satisfaction and frustration of psychological needs as content-specific constructs. They also open new research questions about the processes and dynamics by which experiences of need satisfaction and frustration differently predict pathways of (mal)adjustment in the transition to higher education or to the job market. Additional research is needed to clarify these links.
